# Angiotensin II and Cardiovascular Disease: Balancing Pathogenic and Protective Pathways

**DOI:** 10.3390/cimb47070501

**Published:** 2025-07-01

**Authors:** Ulvi Bayraktutan

**Affiliations:** Academic Unit of Mental Health and Clinical Neurosciences, University of Nottingham, Nottingham NG7 2UH, UK; ulvi.bayraktutan@nottingham.ac.uk

**Keywords:** angiotensin II, renin-angiotensin system, cardiovascular disease, oxidative stress, smooth muscle cell proliferation, angiotensin converting enzyme, senescence, ageing

## Abstract

The renin-angiotensin-aldosterone system (RAAS) is a hormone system that controls blood pressure and fluid and electrolyte balance. Angiotensin II, a key effector, is produced from angiotensin I by angiotensin-converting enzyme (ACE) and exerts its effects through binding to its type 1 (AT1R) or type 2 (AT2R) receptors. AT1R activation promotes vasoconstriction, oxidative stress, endothelial dysfunction, peripheral vascular resistance, and atherosclerosis, all of which substantially contribute to cellular senescence and organismal ageing. Conversely, AT2R activation counteracts these effects by inducing vascular relaxation and attenuating vascular cell proliferation and migration, offering protection against occlusive vascular disease. Additionally, conversion of angiotensin II to angiotensin (1-7) or angiotensin I to angiotensin (1-9) by ACE2 provides further cardiovascular protection by lowering oxidative stress, inflammation, and abnormal cell growth. Bearing these in mind, measures to control angiotensin II synthesis or receptor activity have been at the forefront of antihypertensive treatment. This paper briefly reviews the RAAS and explores the dual role of angiotensin II in promoting disease and mediating vascular protection, with a focus on its impact on ageing and cardiovascular pathology.

## 1. Introduction

The Renin-Angiotensin System (RAS), otherwise known as the Renin-Angiotensin-Aldosterone System (RAAS), is a crucial hormone system that controls fluid and electrolyte balance, systemic vascular resistance, and blood pressure (BP) [1]. Amongst its various components, comprehending peptides and enzymes, angiotensin II (ang II) constitutes the primary effector molecule of the RAAS [2,3]. Overactivation of ang II, due to RAAS dysregulation, plays a key role in vascular resistance as well as sodium and fluid retention and, as a consequence, promotes hypertension [3]. Hence, therapeutic approaches targeting ang II synthesis and activity remain central to managing high BP and minimising its deleterious effects on organs, such as the heart, brain, kidneys, and the vasculature [4].

The pathogenic mechanisms mediated by ang II are multifaceted, involving oxidative stress (OS), endothelial dysfunction, and vascular smooth muscle cell (VSMC) proliferation and migration [5]. It is possible that specific targeting of these mechanisms may mitigate or even help eradicate the deleterious impact of ang II on the above-mentioned organs and potentially extend healthy lifespan in individuals with dysfunctional RAAS or overactive ang II. In addition to its classical roles, ang II is also implicated in cellular senescence and organismal ageing, which impair vascular relaxant responses, trigger or exacerbate hypertensive state, and consequently promote cardiovascular disease [6]. With this in mind, this paper begins with an overview of the RAAS before critically evaluating the molecular and cellular mechanisms underlying the so-called opposing functions of ang II within the context of senescence and cardiovascular disease.

All relevant studies from all years, including both reviews and original articles, have been identified on the PubMed database by a literature search using the key MeSH terms “renin-angiotensin system,” “angiotensin II,” “ang III,” “ang IV,” “angiotensin converting enzyme,” “renin,” “ageing,” “senescence,” “angiotensin II receptors,” “AT1R,” “AT2R,” “ACE2,” “angiotensin (1-7),” “angiotensin (1-9),” and “angiotensin II and cardiovascular disease.” Nottingham University search, ClinicalTrials.gov, and Google Scholar were also used to collect pertinent studies.

## 2. A Brief Overview of the Renin–Angiotensin Aldosterone System

Renin was discovered over a century ago as a powerful vasopressor in the renal cortex. Initially, it was thought to exist only in the circulatory system and play a seminal role in the close control of body fluid homeostasis through the regulation of blood volume and peripheral vascular resistance [1]. However, several members of the RAAS and their breakdown products were later discovered in different parts of the body, including the heart, kidney, and brain [2]. The RAAS is composed of several peptides, enzymes, and their receptors, namely renin, angiotensin converting enzyme (ACE), angiotensinogen, ang I, ang II, ang III, ang IV, angiotensin (1-7), angiotensin (1-9), aldosterone, ang II type 1 receptor (AT1R), and ang II type 2 receptor (AT2R). Angiotensinogen is cleaved by renin to form angiotensin I (ang I), a decapeptide with weak biological activity. Ang I is then hydrolysed by ACE to form ang II, an octapeptide hormone with strong haemodynamic effects [3]. Ang II can be further hydrolysed into ang III and ang IV by other aminopeptidases in plasma and tissue [7]. ACE is a constitutively expressed enzyme predominantly found on the plasma membrane of endothelial cells (ECs), epithelial cells, neuroepithelial cells, and the cells of the immune system, e.g., monocytes and macrophages [8]. While ACE expression is the highest in organs of the respiratory and reproductive systems, such as the lungs, epididymis, and testes, its activity is also detectable in plasma and several other organs, including the kidney, intestine, heart, and brain [9]. Similar to ACE, chymase, a chymotrypsin-like serine protease, can also convert ang I to ang II. Though primarily found in a specific subset of human mast cells, chymase is also expressed by cardiac stromal cells, ECs, and renal vascular smooth muscle cells (RVSMCs). In pathological states affecting the heart, vasculature, and kidneys, chymase-mediated ang II production may serve as an alternative pathway to ACE-dependent generation [10,11].

ACE2 represents a human homolog of ACE. It is a carboxypeptidase that is found either in a soluble form or attached to the cellular membrane in the heart, kidney, intestines, and testis. ACE2 counteracts the classical activity of the RAAS and lowers BP by hydrolysing the vasoconstrictor peptide ang II to ang (1-7), a vasodilatory peptide that induces localised vasorelaxation by binding to Mas receptor [12,13]. This hypotensive effect proposes ang (1-7) as a promising therapeutic target for the management of cardiovascular diseases. Moreover, emerging evidence suggests a broader physiological role for the ACE2/ang (1-7)/Mas axis as its downregulation has been correlated with increased susceptibility to age-related neurodegenerative conditions [14]. In addition, ACE2 also catalyses the conversion of ang I to ang (1-9) that further contributes to BP reduction and exerts protective effects against hypertension-mediated cardiovascular remodelling [15].

As indicated above, the octapeptide ang II can be further metabolised to ang III [(ang (2–8)], a heptapeptide. This is realised by aminopeptidase A, an enzyme that catalyses the cleavage of amino acids from the NH_2_-terminus of proteins [16]. Ang III is primarily recognised for its central effects on BP and is regarded as the main peptide involved in the release of both atrial natriuretic peptide (ANP) and vasopressin, two hormones with opposing effects on cardiovascular function [17]. ANP, a cardiac hormone, lowers BP by stimulating vasodilatation and promoting renal excretion of sodium and water. In contrast, vasopressin, also known as anti-diuretic hormone, increases BP through two distinct mechanisms: by increasing blood volume via enhanced reabsorption of water in the kidneys and by elevating peripheral vascular resistance through vasoconstriction of small arteries [18,19,20]. The effects of ang III are mediated by ang II receptors [21]. Ang III stimulates natriuresis via AT2R activation, especially when AT1R activity is blocked [22]. Additionally, ang III demonstrates analgesic effects in mice and appears to be involved in glycaemic control [23].

**Figure 1:**
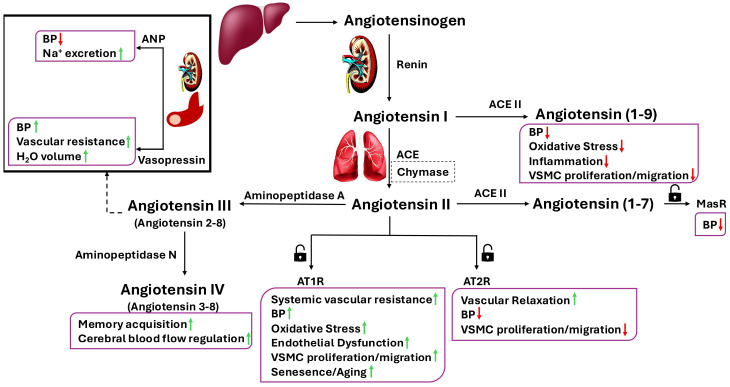
Renin-Angiotensin Aldosterone System and its receptors. Through the enzymatic activity of renin, angiotensinogen is converted to angiotensin I, which is then further metabolised to angiotensin II by ACE or to angiotensin (1-9) by ACE II. Angiotensin II, the active peptide of the renin-angiotensin system, exhibits opposing effects on the cardiovascular system through activation of AT1R or AT2R. Angiotensin II can also be converted to angiotensin (1-7) by ACE II. Both angiotensin (1-9) and angiotensin (1-7) reduce blood pressure (BP). Angiotensin (1-9) exerts its effects by binding to MasR. Angiotensin III, formed by the metabolism of angiotensin II via aminopeptidase A, regulates BP through its effects on ANP and vasopressin. Angiotensin IV, generated from angiotensin III by aminopeptidase N, plays important roles in the modulation of cognitive functions and cerebral blood flow. ACE, angiotensin-converting enzyme; ANP, atrial natriuretic peptide; AT1R, angiotensin II type 1 receptor; AT2R, angiotensin II type 2 receptor; BP, blood pressure; MasR, Mas receptor; VSMC, vascular smooth muscle cell.

Ang III is processed to ang IV [ang (3–8)] by aminopeptidase N. Similar to ang III, the hexapeptide ang IV also exhibits substantially lower pressor activity compared to ang II [24]. Ang IV displays a wide range of activities in the central nervous system (CNS), where it interacts with the AT4 receptor, a binding site implicated in the regulation of memory acquisition and cerebral blood flow [25] (Figure 1). Synthetic small-molecule analogues of ang IV that are able to cross the blood-brain barrier have been successfully developed in laboratory conditions. Both ang IV and its analogues appear to exert beneficial effects on cognitive tasks related to spatial memory, such as object recognition and avoidance. Interestingly, the therapeutic modulation of ang II receptors does not affect the functions of ang IV [26].

The RAAS exists in both intracellular and extracellular forms [27,28]. Accumulating data indicate the importance of local paracrine (tissue-specific) RAAS in the pathogenesis of several organ-specific disorders. In this regard, the role of local RAAS activity in the CNS has attracted attention due to its involvement in major neurodegenerative diseases affecting both cognition and motor systems, such as Alzheimer’s Disease and Parkinson’s Disease [14,29]. It is, therefore, reasonable to suggest that tissue-specific RAAS, acting in concert with the systemic (circulating) RAAS, contributes to the regulation of diverse physiological and pathological phenomena at both local and systemic levels. For instance, through interacting with circulating RAAS, the local RAAS within the kidney has been implicated in the regulation of BP and renal cell growth [30].

## 3. A Brief Overview of the Renin–Angiotensin Aldosterone System Receptors

Ang II binds with high affinity to two pharmacologically distinct G protein-coupled receptors, AT1R and AT2R. Of these, the AT1R is widely distributed in all organs, including the brain, heart, kidney, adrenals, vasculature, lungs, and other peripheral tissues. Most of the classical actions of ang II are mediated by the AT1R [31,32]. These include vasoconstriction, aldosterone synthesis and release from the adrenal zona glomerulosa, renal salt and water retention, stimulation of the sympathetic nervous system, baroreceptor-heart rate reflex, thirst, cell proliferation, fibrosis, inflammation, and OS [33]. Although successive activation of protein kinase C and several growth-related transcription factors, i.e., c-jun, c-myc, and c-fos, modulates the bulk of ang II-mediated trophic effects, ang II can also stimulate mitogen-activated extracellular signal-regulated kinases (MAPKs), Janus kinases (JAKs), and a few members of the signal transducer and activator of transcription (STAT) family through the activation of AT1R [34].

In humans, the AT1R is encoded by a single gene. In rodents, two homologous genes, namely AT1aR and AT1bR, encode AT1R. It is possible that these genes are the end products of a potential gene duplication event [35]. Both AT1aR and AT1bR encode proteins comprising 359 amino acids, with an approximate molecular weight of 41 kDa, and demonstrate approximately 95% sequence homology in their coding regions. Nonetheless, substantial differences are observed within the promoter and untranslated regions of their respective mRNAs [36,37]. Of these, the AT1aR is widely expressed in many tissues, such as the heart, kidney, smooth muscle, liver, and lungs [38]. The expression of AT1bR is more restricted, with predominant localisation in the pituitary and adrenal glands [39]. In accordance with these observations, studies with single and double knockout models in rodents have ascribed the majority of ang II-mediated physiological responses to AT1aR, even though both receptor subtypes exhibit comparable ligand-binding affinities and receptor-effector coupling [36,37].

**Figure 2:**
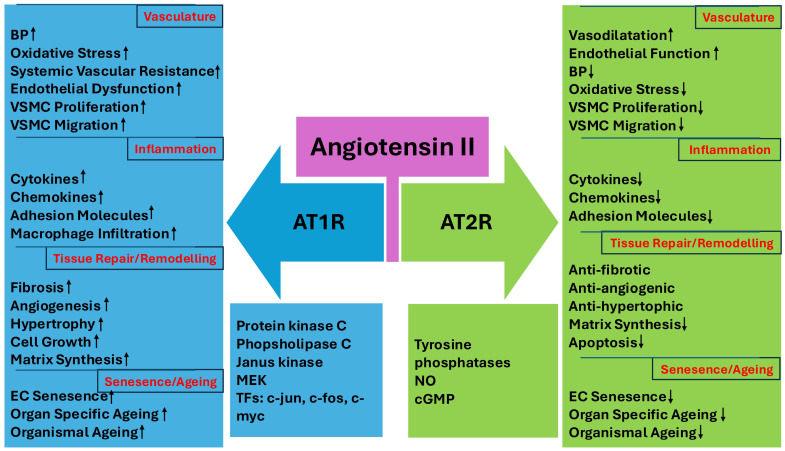
Angiotensin II exerts its functions by binding to AT1R and AT2R. Activation of AT1R leads to increases in BP, oxidative stress, cytokine and chemokine release, EC senescence, and ageing through induction of various signalling pathways and TFs, e.g., protein kinase C, Janus kinases, and c-myc. Activation of AT2R exerts opposite effects to those mediated by AT1R. AT1R, angiotensin II type 1 receptor; AT2R, angiotensin II type 2 receptor; BP, blood pressure; cGMP, cyclic guanosine monophosphate; EC, endothelial cell; TFs, transcription factors; VSMC, vascular smooth muscle cell; MEK, mitogen-activated protein kinase; NO, nitric oxide.

Unlike AT1R, the AT2R is abundantly and transiently expressed in foetal tissues. It is also selectively expressed in a few adult tissues, including vascular endothelium, brain, heart, pancreas, and uterus [40]. However, in several pathophysiological conditions, such as heart failure and tissue regeneration after myocardial infarction, the AT2R expression is transiently elevated [12]. In general, the stimulation of AT2R yields opposite effects to those produced by AT1R (Figure 2). These include vasodilatation, the suppression of OS, inhibition of vascular cell proliferation, and attenuation of inflammation and fibrosis. These effects are primarily mediated through mechanisms involving the release of nitric oxide (NO) and cyclic guanosine monophosphate (cGMP). Hence, the activation of AT2R is implicated in the protection of the heart and kidney against ischaemic injury, fibrosis, and acute infarction [41,42,43].

As alluded to above, the molecular and cellular mechanisms underlying the biological functions of the new members of RAAS have been the subject of extensive investigation for some time. It is now well-established that by acting on the Mas receptor, ang (1-7) exerts effects that counterbalance the vasocontractile, pro-oxidant, pro-fibrotic, and pro-atherogenic effects of ang II [13,44]. Similarly, alamandine [Ala^1^-Ang-(1-7)], a peptide structurally related to Ang-(1-7) and synthesised either directly from it or from angiotensin A (Ang A) by ACE2, exerts cardioprotective effects. Alamandine operates primarily through the Mas-related G protein-coupled receptor D (MrgD), promotes the generation of NO, and, as a consequence, mitigates the deleterious effects of ang II on the cardiovascular system [45,46,47,48].

Ang A is a biologically active derivative of ang II [47,48], capable of binding to both ang II receptor subtypes and inducing vasoconstriction, albeit to a lesser extent than ang II. Crucially, ang A fails to produce any vasodilatory effect even when AT1R is blocked [49]. In translational studies, such as those conducted with rabbits fed an atherogenic diet, vasoconstriction induced by ang A in the abdominal aorta was significantly weaker than that produced by ang II. This finding suggests the presence of a compensatory mechanism in which increased plasma levels of ang A and alamandine may contribute to the modulation of vascular tone under certain pathological conditions [50]. Indeed, clinical observation of significantly higher plasma levels of ang A in patients with end-stage renal disease further supports this notion [51].

In addition to Ang A and alamandine, other downstream metabolites of the RAAS also exhibit distinct receptor affinities and physiological effects. Ang III can bind to and activate both AT1R and AT2R, whereas ang IV binds selectively to the angiotensin type 4 receptor (AT4R), which promotes vasodilation in cerebral, cardiac, and renal vascular beds, enhances cognitive function, increases renal cortical blood flow, and natriuresis [52]. Early studies identified insulin-regulated aminopeptidase (IRAP) as the molecular identity of AT4R and proposed a potential interaction between ang IV and the hepatocyte growth factor signalling pathway through the c-Met tyrosine kinase receptor [53]. Finally, renin itself exerts biological effects through its interaction with a specific receptor, known as the (pro)renin receptor or ATPase H^+^-transporting lysosomal accessory protein 2. Binding of renin or prorenin to this receptor facilitates the conversion of angiotensinogen to ang I and may initiate additional intracellular signalling cascades, thereby contributing to both classical and non-classical RAAS functions [54].

## 4. Angiotensin II and Its Mechanism of Action

Ang II is arguably the most important member of the RAAS. As mentioned above, it increases the synthesis and secretion of aldosterone and elevates vasopressin release by its actions on the CNS. Ang II raises BP by either acting on the Gq protein in VSMCs, which triggers arteriolar vasoconstriction and increases systemic vascular resistance [55], or by acting on the Na^+^/H^+^ exchanger in the proximal renal tubules, which induces renal H^+^ ion excretion and Na^+^ reabsorption, resulting in water retention and an increase in blood volume [56]. It is noteworthy that these mechanisms can act independently or in concert. Besides, ang II overactivity and ensuing hypertension can also trigger positive inotropic and chronotropic effects as well as cardiac enlargement and remodelling [57]. Given the involvement of AT1R in these processes, ACE inhibitors and AT1R blockers (ARBs) are widely used to control hypertension and prevent or delay the progression of cardiac remodelling and heart failure stemming from ang II overactivity [58,59].

Ang II overactivity also promotes a pro-oxidant, pro-inflammatory, and pro-fibrotic state, largely through the generation of reactive oxygen species (ROS) and the activation of redox-sensitive signalling cascades. The AT1R-dependent activation of NADPH oxidase accounts for much of the excessive generation of intracellular superoxide anion (O_2_^•−^), the foundation molecule of all ROS [60]. O_2_^•−^ contributes to vascular dysfunction by reacting with NO, the most potent endogenous vasodilator, and generating peroxynitrite (ONOO^−^) in the process. At high concentrations, ONOO^−^ acts as a cytotoxic compound that damages single DNA strands and depletes key intracellular peptides with antioxidant properties such as glutathione and cysteine [61,62]. Under normal conditions, superoxide dismutases (SODs) metabolise O_2_^•−^ into H_2_O_2_, which is then further catabolised to H_2_O by antioxidant enzymes glutathione peroxidase and catalase [63].

The uncoupling of endothelial nitric oxide synthase (eNOS) due to reduced availability of its cofactor, tetrahydrobiopterin, may also contribute to the increased bioavailability of O_2_^•−^ in pathological conditions associated with RAAS overactivity, such as hypertension and diabetes [64,65]. eNOS constitutes the main source of NO in the vasculature in physiological settings, and although it is a constitutive enzyme, its expression and activity are modulated by several physiological phenomena, including sex hormones, chronological ageing, and shear stress [63]. In contrast to AT1R-mediated effects, amg II binding to AT2R elicits vasodilatation, promotes apoptosis, inhibits cell proliferation and hypertrophy, decreases renal fibrosis, and protects the heart and kidneys against ischaemia-reperfusion injury [41,42,43].

## 5. Ang II in Cell Senescence and Organismal Ageing

In addition to its effects on BP, ang II is also implicated in cell senescence, organ-specific ageing, chronological ageing, and the pathogenesis of age-related degenerative diseases. AT1R-induced telomere attrition, OS, and inflammation appear to be the key mechanisms involved in ang II-evoked cerebral, renal, and cardiac ageing and senescence of various human cells, including glomerular mesangial cells and VSMCs [5,66,67]. The OS theory of ageing posits that the gradual and continuous accumulation of ROS in DNA, proteins, and other macromolecules leads to age-dependent alterations in physiological functions [68,69]. Recent evidence indicates that the overactivation of ang II/AT1R-mediated ROS generation in mice triggers tubular senescence and renal fibrosis along with cardiac dysfunction, coupled with fibrosis and hypertrophy [70,71]. The considerably extended lifespan observed in mice with impaired AT1R and attenuated oxidative damage substantiates this notion and implies that blockade of RAAS or treatments with antioxidants may help minimise or negate organ dysfunction and the overall ageing process initiated by cellular senescence [6,72,73]. Indeed, OS, mimicked by exposure of human brain microvascular ECs or fibroblasts to exogenous H_2_O_2_, induced senescence in these cells, as ascertained by marked increases in senescence-associated β-galactosidase (SA-β-gal) and γ-H2AX staining, along with reduced proliferation rates [74,75,76]. However, antioxidants, such as apocynin, kaempferol, and polyphenol extracts, significantly attenuated not only ang II-induced senescence but also replicative and stress-induced (H_2_O_2_) premature senescence [73,77,78].

Using the INK-ATTAC (p16Ink4a–Apoptosis Through Targeted Activation of Caspase 8) transgenic mouse model, recent studies have demonstrated that a gradual infusion of low-level ang II for 3 weeks produced a small increase in BP, OS, and cardiovascular damage while also inducing ATTAC transgene expression in kidneys [79]. These were accompanied by increases in renal expression of cell cycle proteins (i.e., ATM, p15, and p21) and SASP genes (i.e., MMP3, FGF2, IGFBP2, and tPA). The increased expression of angiopoietin 2 and von Willebrand factor in senescent cells identified them as ECs [80]. The INK-ATTAC model facilitates the conditional elimination of p16Ink4a-dependent senescent cells through the administration of AP20187. The removal of senescent cells by AP20187 prevented physiological, cellular, and molecular responses to ang II and reinforced the prominent role of cellular senescence in the cellular and organismal effects induced by ang II [80].

Accumulating evidence shows that the pervasiveness of cellular senescence is also accelerated by mitochondrial dysfunction [68]. In addition to NADPH oxidase and eNOS uncoupling, ang II also influences intracellular OS by regulating mitochondrial ROS (mtROS). This dramatically impairs mitochondrial energy metabolism and adversely affects survival. In contrast, the activation of mitochondrial function through blockade of RAAS lowers mtROS levels and prevents age-dependent changes in rodents [81]. Considering that ang II is a pleiotropic peptide, the age-retarding and protective effects of RAAS blockade likely involve many distinct mechanisms. The upregulation of Klotho, sirtuins, and peroxisome proliferator-activated receptors, as well as the downregulation of TGF-β, are amongst the mechanisms that activate mitochondrial function [81,82]. The internalisation of AT1R and the regulation of the calcineurin/nuclear factor of activated T cells (NFAT) pathway by AT1R-associated protein (ATRAP) may also contribute to the age-protective effects realised by ang II blockade. Treatments with NFAT-siRNA, which lead to a reduction in VSMC senescence as evidenced by a decline in NFAT transcriptional activity and increases in SA-β-gal staining, OS, and expression of p53 and p21 in wild-type VSMCs, support this finding at the cellular level [83]. The diminished expression of senescence markers in AT1aR-inactivated mice that exhibit increased longevity, possibly due to attenuation of OS and stimulation of prosurvival genes, further corroborates this finding at the organismal level [84].

Senescence is accompanied by a progressive decline in the hormonal profile that affects cardiovascular homeostasis. RAAS becomes dysregulated with age [85]. This dysregulation is partly modulated by the age-related reduction in sex hormones such as oestrogen and testosterone. Oestrogen downregulates ACE and AT1R expression and exerts cardiovascular protective effects by inducing NO production and AT2R-mediated vasodilation. Reduced oestrogen levels in post-menopausal women, on the other hand, stimulate the classical RAAS and, as a consequence, evoke vasoconstriction, inflammation, and oxidative stress [86]. The impact of testosterone on RAAS activity appears complex and variable across tissues and disease states [87]. The interplay between decreased levels of sex hormones and RAAS dysregulation during senescence emphasises the importance of this phenomenon in the pathophysiology of age-related cardiovascular disorders and warrants future studies focusing on sex-specific therapeutic strategies targeting RAAS components [88]. Taken together, these findings pinpoint the relationship between ang II and the ageing process and provide evidence for future strategies aiming to delay the vascular ageing process and age-related vascular disease formation.

## 6. Angiotensin II in Cardiovascular Disease

Despite its well-documented effects pertaining to arterial vasoconstriction and BP regulation, ang II in fact executes most of its cellular functions, such as the induction of VSMC, fibroblast, and cardiomyocyte proliferation and migration, independent of these effects. The overactivation of ang II contributes to increased inflammation and OS. These, in turn, damage the endothelial lining of blood vessels and promote atherosclerosis, characterised by deposition of LDL-cholesterol, formation of foam cells, and infiltration of macrophages and other white blood cells into the vascular endothelium [89,90]. Taken together, these processes can seriously compromise vascular structural and functional integrity, narrow the vascular lumen, and may give rise to cardiovascular remodelling, myocardial infarcts, and stroke.

**Figure 3:**
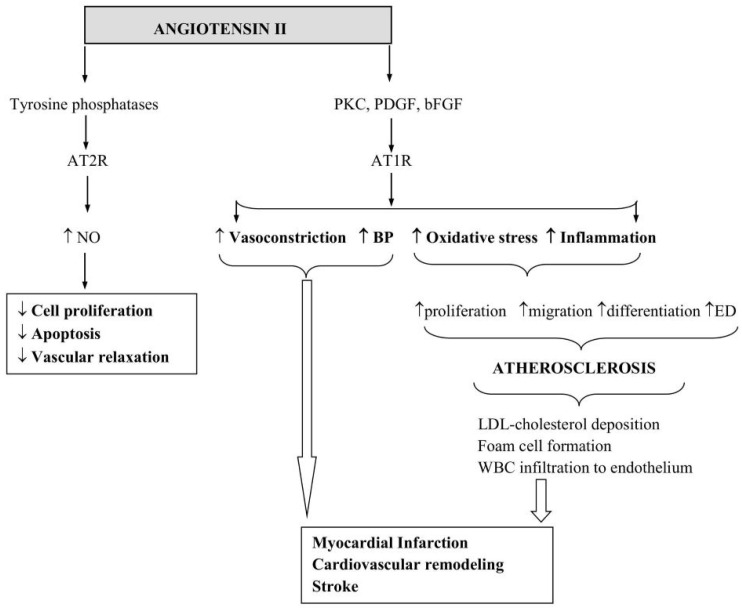
Angiotensin II exerts both cardiovascular protective and destructive effects. By binding to its type 2 receptor (AT2R), ang II triggers the release of nitric oxide and, consequently, promotes vascular relaxation while suppressing cell proliferation and apoptosis. By binding to its type receptor (AT1R), ang II increases BP and evokes vasoconstriction, oxidative stress, and inflammation. Elevations in oxidative stress and inflammation stimulate vascular cell proliferation, migration, and differentiation, leading to endothelial dysfunction and atherosclerosis. Through atherosclerotic disease formation and its direct effects on vascular tone and blood pressure, ang II contributes to the development of myocardial infarction, cardiovascular remodelling, and stroke. BP, blood pressure; NO, nitric oxide; ED, endothelial dysfunction; PKC, protein kinase C; PDGF, platelet-derived growth factor; bFGF, basic fibroblast growth factor.

As indicated above, the AT1R modulates most of the well-known vascular effects of ang II through the induction of several signalling pathways and growth factors, such as protein kinase C, phospholipase C, platelet-derived growth factor, and basic fibroblast growth factor [31,32,91]. In contrast, the AT2R mediates the cardiovascular protective effects of ang II through the induction of tyrosine phosphatases, which suppress cellular proliferation and apoptosis [92] (Figure 3). Studies employing cell culture models or transgenic mice lacking the AT2R gene confirm the role of AT2R in mediating anti-hypertrophic and anti-fibrotic effects of ang II [93,94]. Importantly, these findings also provide evidence for why cell lines that express both ang II receptor subtypes may display opposing activities when subjected to ang II. Studies using rat coronary microvascular EC (CMEC) and aortic ECs suggest that local ang II concentrations determine which receptor subtype is activated [95,96]. In this context, the detection of relaxant responses in endothelium-intact vessels and contractile responses in endothelium-denuded vascular rings highlights the efficacy of ang II in modulating the NO release, a key endothelium-derived relaxing factor [95,96,97]. Within the endothelium, NO is synthesised from the amino acid L-arginine by the enzymatic activity of eNOS, a Ca^2+^/calmodulin-dependent enzyme. Once generated in sufficient quantities, NO suppresses both the proliferation and migration of VSMCs and is, therefore, regarded as an important anti-atherogenic molecule. So, ang II may indirectly mitigate atherogenesis by increasing NO bioavailability [63]. However, by also promoting OS and an inflammatory state, ang II also induces vascular cell proliferation, migration, and differentiation, and as a consequence triggers atherosclerosis [60]. Albeit a constitutively expressed enzyme, various physio-pathological processes, notably cell proliferation and genetic hypertension, can alter the expression and functionality of eNOS [95,98,99]. Two seminal studies investigating the potential interactions between NO and ang II within the context of endothelial cell growth, a key event in endothelial regeneration after injury and prevention of atherosclerotic disease progression, reported over 2-fold increases in basal nitrite levels (an index of NO generation) as well as eNOS mRNA and protein expression in proliferating versus resting (fully confluent) rat aortic ECs and CMECs. The induction of similar increases with ang II in growing and resting rat aortic EC nitrite production suggests a direct or indirect role for ang II in the stimulation of eNOS activity [95,96]. The complete cessation of nitrite production in these cells with L-NNA, a NOS inhibitor, provides evidence for this hypothesis and implicates a Ca^2+^/calmodulin-dependent signalling pathway in ang II-mediated nitrite production [100,101]. In relevant studies, acquisition of substantially greater vascular relaxant responses in endothelium-denuded vascular rings co-treated with ang II and an ARB (valsartan) indicates that the bradykinin/NO pathway plays a role in ang II/AT2R-mediated vascular relaxation [95,96,102,103].

In addition to ECs, ang II also evokes the migration and proliferation of VSMCs, which represent the key events in atherosclerosis [104,105,106]. The attenuation of ang II-induced SMC migration and proliferation by an AT1R blocker (losartan), a SOD mimetic (MnTBAP), and an NADPH oxidase inhibitor (apocynin) highlights the pivotal roles of AT1R and O_2_^•−^ in these processes [60,63,105]. These findings also highlight the importance of the body’s inherent anti-oxidant defence systems to successfully suppress atherosclerotic disease progression [107,108]. Ironically, while O_2_^•−^ contributes to OS and vascular pathology, it also acts as an important signalling molecule at physiological levels and is a prerequisite for normal CMEC growth. Inhibition of cell proliferation by an inhibitor of NADPH oxidase supports this role [109]. The inefficacy of other ROS-generating enzymes, such as xanthine oxidase or cyclooxygenase, in influencing CMEC growth signifies the importance of the NADPH oxidase system in generating biologically relevant quantities of O_2_^−^ [109]. NADPH oxidase is composed of several cytosolic subunits, namely p40-phox, p47-phox, and p67-phox, and two membrane-bound components, i.e., p22-phox and gp91-phox that make up cytochrome b558 and account for enzyme activity and stability as a whole [110,111]. A previous study probing the reciprocal relationship between CMEC growth and the expression and activity of NADPH oxidase has shown significant increases in p22-phox mRNA and protein expression, NADPH oxidase activity, and O_2_^−^ generation in proliferating versus resting CMEC [96]. Furthermore, the targeted inhibition of NADPH oxidase activity in CMECs through antisense p22-phox cDNA transfection has led to a significant decrease in cell growth without affecting their viability [112].

## 7. Impact of RAAS Modulation on Disease Outcome

As discussed above, various physio-pathological phenomena, such as ageing and hypertension, lead to a marked, time-dependent increase in ang II levels, which exacerbate both local and systemic vascular injury, accentuating the therapeutic value of ACE inhibitors and ARBs in these individuals [113,114]. In accordance with this statement, the incidence of atherosclerosis and myocardial infarction appears to be markedly lower in hypertensive rats treated with these agents [115,116]. Furthermore, compared to agents targeting BP alone, such as diuretics and smooth muscle relaxants, agents targeting RAAS have proven to provide much better protective effects on cardiovascular structure and function [95]. In support of this, a large meta-analysis investigating cardiovascular outcomes in diabetic patients for at least 12 months revealed that treatments with ACEIs (32,827 patients) but not ARBs (23,867 patients) significantly reduced the risk of all-cause mortality, cardiovascular death, and major vascular events, e.g., myocardial infarction. These findings consolidate the use of ACEIs as first-line in this population [117].

Notably, the relevance of the RAAS has been further highlighted during the COVID-19 pandemic, as SARS-CoV-2, the virus responsible for COVID-19, enters host cells via the ACE2 receptor. This interaction perturbs the balance between ang II and angiotensin-(1-7). Considering that the ACE2/angiotensin (1-7)/MAS axis counteracts the negative effects of the RAAS [112], the disruption of this balance may potentially exacerbate inflammation, endothelial dysfunction, and vascular injury. Consequently, understanding and modulating RAAS activity through ACEIs and ARBs has garnered significant interest, not only for cardiovascular protection but also as a potential therapeutic strategy for the management of COVID-19-related complications [118,119].

## 8. Conclusions

In conclusion, ang II modulates a wide range of cellular processes, including cytoskeletal organisation, behaviour, function, and senescence, which may contribute to the initiation and progression of cardiovascular diseases and help determine their clinical outcome. The continued, clinical efficacy of ACEIs and ARBs in managing hypertension, together with their protective benefits on target organs, emphasises the importance of research into RAAS [120]. Furthermore, accumulating evidence illustrates that ang II blockade may hold promise beyond cardiovascular protection, thereby warranting further research into its potential to delay the physiological ageing process and mitigate the problems associated with ageing-related chronic diseases to improve health span.
